# Metabolomics as an Emerging Tool for the Study of Plant–Pathogen Interactions

**DOI:** 10.3390/metabo10020052

**Published:** 2020-01-29

**Authors:** Fernanda R. Castro-Moretti, Irene N. Gentzel, David Mackey, Ana P. Alonso

**Affiliations:** 1 BioDiscovery Institute, University of North Texas, TX 76201, USA; Fernanda.Moretti@unt.edu; 2 Department of Biological Sciences, University of North Texas, TX 76201, USA; 3 Department of Plant Pathology, The Ohio State University, Columbus, OH 43210, USA; gentzel.3@osu.edu; 4 Department of Horticulture and Crop Science, The Ohio State University, Columbus, OH 43210, USA; mackey.86@osu.edu

**Keywords:** gas-chromatography, liquid-chromatography, mass spectrometry, microbe, pathogen, plant, primary metabolites, specialized metabolites

## Abstract

Plants defend themselves from most microbial attacks via mechanisms including cell wall fortification, production of antimicrobial compounds, and generation of reactive oxygen species. Successful pathogens overcome these host defenses, as well as obtain nutrients from the host. Perturbations of plant metabolism play a central role in determining the outcome of attempted infections. Metabolomic analyses, for example between healthy, newly infected and diseased or resistant plants, have the potential to reveal perturbations to signaling or output pathways with key roles in determining the outcome of a plant–microbe interaction. However, application of this -omic and its tools in plant pathology studies is lagging relative to genomic and transcriptomic methods. Thus, it is imperative to bring the power of metabolomics to bear on the study of plant resistance/susceptibility. This review discusses metabolomics studies that link changes in primary or specialized metabolism to the defense responses of plants against bacterial, fungal, nematode, and viral pathogens. Also examined are cases where metabolomics unveils virulence mechanisms used by pathogens. Finally, how integrating metabolomics with other -omics can advance plant pathology research is discussed.

## 1. Introduction

### Plant Metabolism is A Complex and Dynamic Process

**Figure 1:**
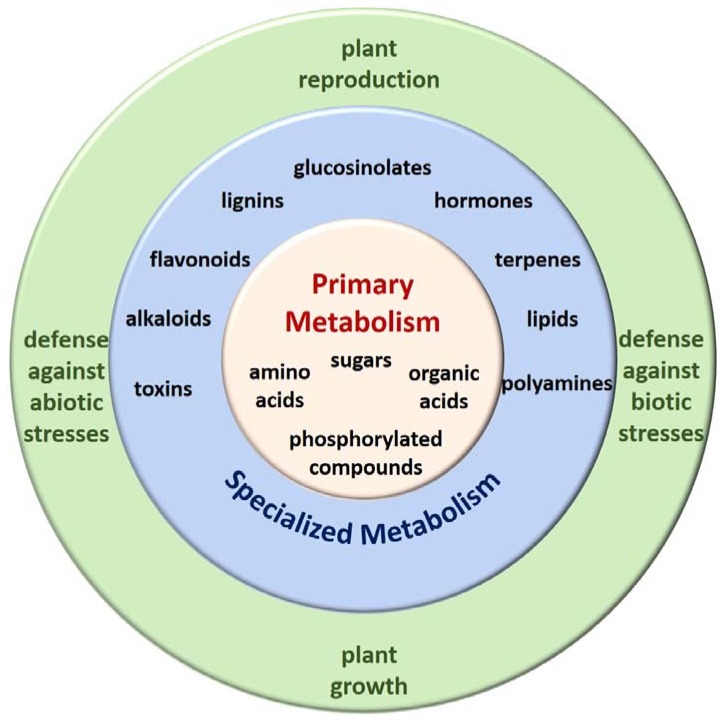
Plant metabolism. Primary metabolism (yellow) revolves around critical physiological compounds such as amino acids and sugars. Secondary metabolism (blue) utilizes central metabolites as building blocks for the biosynthesis of specialized compounds such as flavonoids, toxins, and lipids that have various functions (green), including plant growth and defense against stresses.

Often described as natural chemists, plants can produce thousands of unique metabolites that serve to attract pollinators, repel herbivores, combat microbial pathogens, and provide protection from environmental stresses [1]. This propensity to biosynthesize a seemingly endless array of diverse molecules has made plants a staple in natural medicine and the pharmaceutical industry alike, as these molecules have activity against diseases such as malaria, Alzheimer’s, and cancer [2,3,4,5]. Plant metabolism can be divided into two general categories: primary and specialized metabolism [6]. Primary metabolism involves compounds critical to growth, development and reproduction of the plant, whereas specialized metabolism encompasses compounds needed for the plant to successfully cope with abiotic and biotic stresses (Figure 1) [5,6,7]. These classes of metabolism are intrinsically linked; the metabolites of primary metabolic pathways, such as glycolysis, the pentose–phosphate pathway, and the tricarboxylic acid cycle, also serve as building blocks for secondary metabolic pathways. Amino acids, for example, participate not only in nitrogen assimilation, but also as precursors for a number of specialized compounds including hormones, and pigments. 

Due to their sessile nature, plants rely heavily on chemical defense against biotic and abiotic stresses. Therefore, plant metabolism is a dynamic process that responds to external stimuli. Environmental changes such as light quality, water stress, or temperature have been shown to impact metabolism [8,9,10,11,12,13]. This review focuses on the roles of primary and specialized metabolism in defense responses or disease progression during plant–pathogen interactions.

## 2. The Evolution of Metabolomics in the Study of Plant–Microbe Interactions

Plant diseases account for a significant portion of crop losses worldwide, in addition to those caused by abiotic stresses such as drought or saline soils [14]. As reviewed by Reddy et al. (2009), crop losses to viruses alone can be up to 82% in banana and 100% in cocoa [15]. Bacterial, fungal, viral, and parasitic nematode diseases resulted in an estimated $26 billion loss from 2010 through 2014 in the United States soybean crop alone [16]. In a comprehensive study of corn grown in the United States and Ontario, Canada, Mueller, et al. (2015) estimated a 11.8% loss due to disease over a span of four growing seasons [17]. Severe losses due to pathogen outbreaks can have significant societal impact, such as the Irish potato famine in the 1840s due to the fungal pathogen *Phytophthora infestans,* or the widespread loss of sweet corn in the US during the early 20th century due to the bacterial pathogen *Pantoea stewartii* subsp. *stewartii* [18,19]. Losses such as these helped drive research towards understanding how plant pathogens cause disease and how to prevent such outbreaks in the future. The endeavor to develop resistant varieties against these diseases has largely relied on breeding efforts to introduce resistance alleles to elite lines [20]. While effective, at least in the short-term, this strategy typically does not elucidate the mechanism behind disease development for a given system. To achieve this understanding, plant pathologists have instead relied on phenotypic and molecular assays to assess symptom development, gene expression, protein interactions, and the like [21]. By utilizing model plant organisms such as *Arabidopsis thaliana*, tomato, and maize, our understanding of many plant diseases has been advanced [22]. Finally, plant–pathogen studies that integrated observations of host metabolism have paved the way to a better understanding of plant disease mechanisms [23,24,25,26,27]. 

### 2.1. Early Plant Pathogenic Studies

Metabolomics as we know it today is actually a relatively new endeavor in plant pathology research. While researchers have long recognized the importance of individual metabolites during plant diseases, only recently have plant pathologists begun to embrace more global analyses offered by metabolomics. Traditional methods to study plant disease have relied on phenotypic analyses such as comparisons of symptom development between susceptible and resistant varieties, and various molecular readouts of plant defenses such as reinforcement of the plant cell wall by callose deposition at the site of infection [28,29], production of reactive oxygen species (ROS) as antimicrobial and signaling molecules [30], and the secretion of other antimicrobial compounds [31]. The importance of plant hormones is firmly established in plant disease interactions, with certain hormone signatures correlated to attack by a biotrophic or necrotrophic pathogen [32,33]. All of these are outputs of plant metabolism, however, which provokes the question of how these responses fit into the larger scheme of plant physiology on the metabolite level.

### 2.2. How the -Omics Have Contributed to Plant Pathogen Research

Prior to the advent of metabolomics, the development of genomics, transcriptomics, and proteomics contributed greatly to our understanding of plant diseases and the mechanisms that determine whether a pathogen successfully obtains nutrients and evades plant immunity. Genomics studies analyzing the genetic architecture of both plants and pathogens have been useful to monitor how the organisms adapt to disease pressure [34,35]. Transcriptomic studies have given insight as to what host genes are manipulated by pathogens in a disease setting, or are reprogrammed for a successful defense response. Sugarcane mosaic virus (SCMV), which is a major concern for Chinese maize growers, was shown in a transcriptomics study to drastically downregulate photosynthesis genes consistent with the chlorotic lesion phenotype [36]. Additionally, this research group assessed translational responses via ribosome profiling. Interestingly, two transcriptionally downregulated phenylpropanoid biosynthesis enzymes—4-coumarate coenzyme a ligase (4LC) and phenylalanine ammonia lyase (PAL)—were upregulated on the translational level [36]. *Pantoea stewartii*, which is a significant pathogen of maize in the north-central and eastern US, similarly downregulates the abundance of transcripts of photosynthesis genes but also induces the expression of numerous phenylpropanoid metabolic enzymes, including 4LC and PAL [26]. Studies such as these reveal the complex nature of plant responses to pathogens, and highlight the need to examine metabolites directly to better characterize the pathosystem. Proteomic studies can yield important information on pathogen host targets, interactors, and elicitors of disease from the pathogens [37]. Researchers analyzed the impact of one such elicitor—the proteinaceous ToxA secreted by the fungal pathogen *Pyrenophora tritici-repentis*—on the wheat proteome and discovered a decrease in the levels of photosystem II supercomplexes, which putatively would increase the levels of ROS and drive symptom development [38].

### 2.3. Advances in Plant Pathology Using Metabolomics Approaches

**Figure 2:**
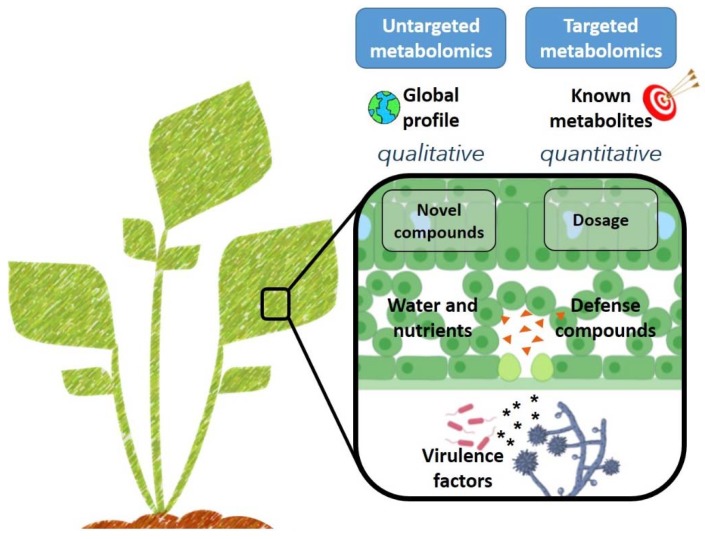
Metabolomics as a tool to unveil plant–pathogen interactions. The untargeted approach is qualitative and gives a global profile of many unknown metabolites in a sample. The targeted approach is quantitative and more specific, as it aims for a determined class of known compounds.

As host resistance genes and virulence targets of plant pathogen were identified, the need arose to investigate more fully the phenotypes associated with these interactions. Metabolomics can provide a snapshot of plant metabolism during development and in response to a wide range of biotic and abiotic stimuli, including environmental or nutritional stresses [39,40,41,42,43,44]. Metabolomics studies are often classified in terms of the analysis completed. In targeted metabolomics, a finite list of compounds are selected for analysis, whereas untargeted analyses scan for an undefined number of unique features [45] (Figure 2). In either case, the scope of identifiable compounds is restricted by experimental parameters, including extract preparation and instrumentation [46]. For a given biological sample, different metabolites will be extracted depending on the chemical properties of the extraction solvent. Water, the universal solvent, was shown to efficiently extract a range of primary metabolites from plant leaves, including amino acids, sugars, organic acids (OAs), and phosphorylated compounds (PCs) [47]. Specialized metabolites can be extracted from plant matter more efficiently with methanol, for example [48]. In addition to solvent choice, a second consideration for characterizing the metabolite profile of plant–pathogen interactions relates to the instrumentation utilized to identify and/or quantify compounds of interest. Gas chromatography-mass spectrometry is often employed to generate large qualitative data sets from volatilized samples, whereas liquid chromatography-tandem mass spectrometry is frequently used to create small quantitative data sets on minimally-processed samples [42,45].

Metabolomics has been particularly useful in the natural products arena to discover novel compounds that may be associated with the bioactivity of plant extracts used for human health and disease treatment [49]. The same concept can be used for understanding plant responses to pathogens, whether in a compatible interaction where disease progresses, or in an incompatible interaction where the pathogen fails to cause disease. For example, in a recent study of soybeans infected with the oomycete pathogen *Phytophthora sojae*, metabolomics analysis revealed many sugars and secondary metabolites that differentially accumulated in resistant plants compared to the susceptible variety, hinting that these molecules may play a role in defense [50]. In another study on citrus canker, NMR analysis showed that transgenic expression of sarcotoxin—an antimicrobial peptide effective against the disease—led to a reduction of pathogen-induced metabolite accumulation in infected plants [51]. Due to the universality of primary metabolite structures and the broadly conserved structures across specialized metabolites, metabolomics is a particularly effective way to study plant pathogen interactions across plant varieties and pathogen races. Because genomic resources are not required, researchers can use metabolomics to study nearly any system, including novel species.

## 3. Getting to Know the Enemy

### 3.1. Plant Pathogens Tools for Attack

Plants can be infected and colonized by many types of microorganisms, including bacteria, fungi, viruses, and nematodes, which coexist with and parasitize plants through diverse lifestyles [52]. Necrotrophic plant pathogens proliferate by feeding on dead plant cells produced by secretion of enzymes that degrade plant cell walls and toxins that target various host processes [53]. Biotrophic pathogens obtain nutrients from living plant cells and usually target a narrow range of species, growing systemically and sometimes even inconspicuously [54]. This group of pathogens is comprised by viruses, fastidious bacteria, fungi that cause rust and powdery mildew, oomycetes and nematodes [55]. Combining both lifestyles, hemi-biotrophic pathogens have a biotrophic phase where structures are formed for nutrient acquisition with a later necrotrophic stage which results in their host cell’s death [56,57]. The life-cycle of many pathogenic fungi and bacteria include survival as facultative saprophytes and/or endophytes as well as hemi-biotrophic pathogenesis [55]. Each survival style affects how pathogens orchestrate their attack. 

**Figure 3:**
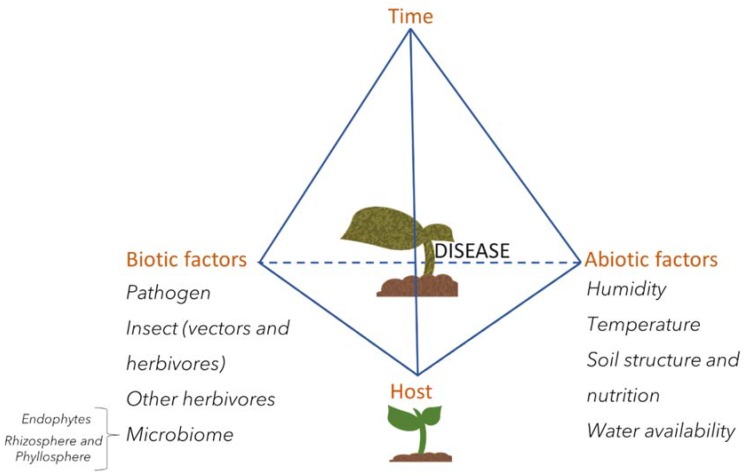
Disease factors. Updated disease triangle into a pyramid, representing abiotic and biotic factors that interact with the pathogen and host to determine disease development as a function of time.

To cause disease, however, the pathogen needs to find ideal conditions. This concept was previously described as the disease triangle, where tendency to the disease state depends on three factors: The pathogen, the host, and the environmental conditions [58]. Advances in plant biology and microbe-interactions are now adding more factors related to disease occurrence, revealing an extremely complex system beyond just the host and its invader. Abiotic factors such as climate, soil nutrient availability, water supply, and circadian rhythms, as well as biotic factors, such as the presence of insects, other pathogens in the same or distal portions of the plant, and the plant’s microbiome, all combine to influence the host–pathogen interaction as a function of time (Figure 3). Understanding the mechanisms of pathogenesis and host defense will lead to enhancement of plant defense across environmental conditions and, ultimately, in the field.

Metabolomics, a tool widely used in pharmaceutics and other bio-analytical procedures, is now becoming indispensable in the study of plant–pathogen interactions. Metabolites perform diverse roles in plant–pathogen interactions, including surveillance against pathogen attack, signal transduction, enzyme regulation, cell-to cell signaling, and anti-microbial activity [59]. The approach can be used to detect a series of metabolites related to infection, such as molecules secreted by pathogens during colonization [60], or amino acids and sugars whose production is induced or mis-localized in the host to enhance pathogen growth. *Plasmodiophora brassicae*, for example, takes over the control of cytokinin synthesis in its host and induces gall formation in infected members from the Brassicaceae family, causing clubroot disease [61]. Amino acids from potato root exudates stimulate spore germination of the pathogenic fungus *Spongospora subterranean* [62]. Tetrose and pentose sugar alcohols accumulated due to altered host amino acid and sugar metabolism in plants of the Rosaceae family infected with *Gymnosporangium asiaticum* [63]. Since metabolites are the intermediaries and products of biochemical pathways coordinated by genes and their related products [64], monitoring their levels can complement and corroborate transcriptomic and/or proteomic data on plant–pathogen interactions, thus unveiling pathogen attack mechanisms. Changes in messenger RNA and proteins are studied by transcriptomic and proteomic analysis, respectively. The products of these gene-centered mechanisms can be also regulated by metabolites [65]. Thus, the integration of metabolomics data with other omics data [64] adds additional layers of information to studies of plant–pathogen interactions, including identification of metabolites that have antimicrobial actions [66], metabolomic profile differences between infected and non-infected plants [67,68,69], and pathogenic requirements for infection and colonization [68,70].

### 3.2. How Metabolomics can Contribute to Understanding Plant Pathogen Attack Methods

Effectors are molecules secreted by pathogens during infection that perturb host processes in order to inhibit defense or promote the availability of water and nutrients [71]. Effectors are often proteins [72], but also include a variety of non- protein metabolites [73]. The polyketide phytotoxin coronatine (COR), that is secreted by *Pseudomonas syringae*, provokes metabolic imbalance in infected plants by functioning as a potent ligand for the jasmonic acid (JA) receptor, COI1, and also through COI1-indpendent activity [74,75]. Additionally, COR and other effectors facilitate pathogenic entrance to the interior of host tissues and cells [76]. Metabolomics can identify the metabolic breakdown caused by effectors and their producers. For instance, numerous effectors converge on influencing the balance between host salicylic acid (SA)- and JA-signaling. COR and bacterial protein effectors variously target JA-signaling [77,78,79,80]. An integrated study on maize and the causal agent of corn smut (*Ustilago maydis*) indicated that effectors from this pathogen suppress the biosynthesis of salicylic acid [81,82]. Toxins are also a tool used by pathogens for attempting infection and colonization. While molecular genetics has proved essential for detecting microbial toxin function in pathogenesis [83], metabolomics can characterize and identify them. For instance, an integrated approach characterized secondary metabolites in *Fusarium culmorum*, using GC-MS and UPLC-MS/MS [84]. Rubrofusarin and other toxins were identified along with terpenes and other secondary metabolites [84]. *Fusarium* sp. are known for causing disease in cereal crops, producing a wide range of toxins while doing so [85,86]. Due to health risks caused by mycotoxins, LC-MS/MS methods have been developed to track those toxic molecules in beverages whose sub products might have been infected by plant–pathogenic fungi [87,88]. In the following sections, we discuss examples of biological markers related to infection that were identified by metabolic analyses. Table 1 summarizes attack and defense molecules cited in this review.

#### 3.2.1. Necrotrophic Arsenal

*Rhizoctonia solani* is a soilborne basidiomycete with a broad host range [89]. As most necrotrophic plant pathogens, *R. solani* produces many toxic compounds that promote infection by causing necrosis and negatively interfering with the host immunity [90]. The effects of this necrotrophic pathogen and its toxin, phenylacetic acid, on maize were evaluated through metabolomics. This study revealed that the susceptible interaction between the fungal invader and its host is tissue-specific, showing that a broad-host range pathogen can produce selective toxins for attacking specific hosts. Also, it was shown that L-glutamate levels vary among resistant and susceptible tissues in infected plants, indicating that pathogen- and host-manipulation of glutamate metabolism may underlie the ability of plant cells to remain viable to resist necrotrophic pathogenesis [91,106].

#### 3.2.2. Biotrophic Elegance

Biotrophic pathogens are known for having evolved sophisticated parasitism mechanisms [54]. Huanglongbing is a devastating disease in citrus, caused by *Candidatus Liberibacter*, a fastidious bacterium limited to the phloem vessels of infected plants [107]. So far three spp. have been found: Ca. *Liberibacter americanus*, Ca. *Liberibacter asiaticus,* and Ca. *Liberibacter africanus* [108], which can be introduced into the plant by two psyllid vectors: *Diaphorina citri* and *Trioza erytreae* [109]. *T. erytreae* is endemic to Africa, but was recently detected in some European countries [110], posing a serious threat to citrus production in these regions. Huanglongbing is a complicated pathosystem to study and has also proven difficult to manage in the field; no treatments or resistance are known for the disease [111]. Metabolomics studies have unveiled crucial information for fighting this disease, also known as greening. Metabolic profiling of orange juice gave key information on the differences between infected and non-infected plants. Among them was a higher abundance of phenylalanine in the presence of the bacteria, which indicated an imbalance on the phenylpropanoid pathway, a major route for plant defense biosynthesis [68]. Gas chromatography coupled to mass spectrometry (GC-MS) was used to compare the metabolic profile of orange plants infested by *D. citri* and infected by *Ca. Liberibacter* [112]. Interestingly, infected leaves presented lower levels of ferulic acid, an important compound related to lignin production and cell wall formation [113,114]. Manipulation of the host by pathogen effectors is likely, as the expected plant response was higher levels of these defense-related metabolites. 

Plant nematodes are obligate soilborne pathogens that infect plant roots. They have evolved sophisticated mechanisms for parasitism, with different feeding stylets and effector secretion for host manipulation [115,116]. They can be roughly divided into three main categories, according to the symptoms induced in their hosts: i) Cyst nematodes are associated with the sedentary genera *Heterodera* and *Globodera*; ii) root-knots are mostly caused by the sedentary species of *Meloidogyne* genus; and iii) lesions are mainly associated with the migratory endoparasitic genus *Pratylenchus* [117]. To succeed in root penetration and nutrient uptake, nematodes insert their stylets into the host roots. This invasive process can elicit host defense responses. *Heterodera schachtii*, which attacks various plant species [118], induces the formation of multinucleated cells (aka syncytia) in susceptible hosts [119]. These so-called “giant cells”, which are produced through endoreduplication, result in major cellular imbalance for the host [120,121]. Hormone profiling showed that infected roots had increased production of ethylene and jasmonic acid, while abscisic acid and gibberellin were less abundant [122]. Additional metabolomics analyses revealed that *H. schachtii* infection influences amino acid production [123,124,125]. Arginine and proline have been shown to play a major role in *H. schachtii* attack on Arabidopsis, corroborating results from gene expression and histochemical assays [126].

#### 3.2.3. Semi-Biotrophic Dual Armament

*P. syringae* has served as a useful model for the study effector-mediated suppression of host immunity [127]. Manipulation of host hormone levels is a key virulence mechanism of this bacterium. In addition to manipulation of SA- and JA-signaling by COR, previous studies have shown that *P. syringae* interferes with host abscisic acid levels to increase susceptibility [128]. Additionally, targeted LC-MS/MS analysis from another study demonstrated that *P. syringae* produces indole-3-acetic acid (auxin), a major plant signaling hormone, which indicates further host manipulation [129]. An integrated study showed that daidzein production is inhibited by *P. syringae* [27]. Daidzein forms part of the isoflavonoid defense build-up [130] and its biosynthesis is regulated by the enzyme 2-hydroxyisoflavone dehydratase (GmHID1). Inoculated soybean plants were more susceptible to infection when *gmhid1* was silenced; HPLC was used to confirm the isoflavone abundance in leaves, validating gene expression results [27]. *Colletotrichum* sp. are also semi-biotrophic pathogens [57]. Interestingly, a genomic and transcriptomics study revealed that different effectors are secreted in each biotrophic or necrotrophic lifestyle [131]. Moreover, more recent integrated research with metabolomics showed that terpenoid production is related to *C. higginsianum* pathogenesis, the causal agent of antrachnose in a wide spectrum of crops [132,133].

## 4. Plant Immunity and Sources of Resistance

### 4.1. Plant Defense Mechanisms

Plants attempt to preclude access of microbes to the interior of plant tissues. Preformed barriers, such as the waxy cuticle, and active closure of stomatal pores restrict access of some potential pathogens. Pathogens that are able to overcome these barriers gain access to the apoplast, which is the extracellular spaces within a plant tissue. As a metabolic hub for many plant physiological processes, including the routing of sugars produced by photosynthesis to the phloem, the evaporation of water during transpiration, and the exchange of carbon dioxide and oxygen during photosynthesis [134], the apoplast affords pathogens close access to nutritional sources and water needed for their proliferation. 

Potential pathogens that enter the apoplast must engage with the complex network of perception, signaling, and response outputs of the plant innate immune system [127,135]. Perception is achieved by plasma membrane-localized pattern recognition receptors (PRRs) that recognize specific, conserved microbial features, including bacterial flagellin and fungal chitin, which are collectively termed pathogen-associated molecular patterns (PAMPs) [136]. PAMP-triggered immunity (PTI) consists of diverse cellular responses, including reactive oxygen and calcium bursts, MAPK (mitogen-activated protein kinase) signaling, plant hormone responses, transcriptional reprogramming, and cell wall fortification [127,137,138,139,140] that collectively inhibit pathogen proliferation [140,141,142,143]. Successful pathogens deploy virulence effectors that produce effector-triggered-susceptibility by overcoming these basal immune responses [144,145]. The cellular targets and mechanism of action of these defense suppressing effectors is widely varied and includes, for example, degradation of PRR proteins [146] and suppression of MAPK activities [147]. 

While the ability of effectors to suppress host immune defenses is well established, their roles in other aspects of disease development, including causing water-soaking and nutrient acquisition, are only emerging. Water-soaking is the macroscopic accumulation of fluid in the apoplast and a hallmark symptom of diseases caused by fungal, oomycete and bacterial plant pathogens. The importance of water in the disease triangle, which considers the environment in addition to host and pathogen, contributes to an understanding of why rain or high humidity are harbingers of plant disease outbreaks. Bacterial virulence effectors have recently been implicated in the induction of water-soaking [148]. Additionally, pathogens must obtain nutrients to proliferate and cause disease. Examples of microbial effectors that promote nutrient acquisition include transcription activator-like effectors from *Xanthomonas* and *Ralstonia* that induce expression of plant SWEET sugar transporters or cell wall degrading enzymes, an effector that functions as an organic acid transporter, and effectors and toxins that disrupt membrane integrity of plant cells [149,150,151,152,153,154]. The role of host metabolism in effector-induced conversion of the apoplast into a nutritive and wet environment is a prime area of future investigation.

Despite the importance of effector-mediated defense suppression and nutrient and water acquisition, virulence effectors also serve as the elicitors of another layer of plant defense, effector-triggered-immunity. Plant resistance (R)-proteins, upon direct or indirect recognition of a pathogen effector, induce robust plant defense that often includes the hallmark hypersensitive response, a type of programmed cell death, at the site of infection [155]. The activation of an R-protein, or the downstream signaling, can also be inhibited by other pathogen effectors, and so continues the molecular arms race between pathogens and plants. 

### 4.2. Metabolomics and Plant Defense

Plant defense metabolites are wide and varied, as would be expected for a stationary organism subject to a plethora of stresses, including microbial threats. To better understand how metabolites function in plant–pathogen interactions, it is useful to categorize the molecules based on, for example, their structure, biosynthesis, localization, or function. Here we describe a few categories involved with plant defense that have been studied using metabolomics techniques. 

#### 4.2.1. Phytoalexins and Phytoanticipins

As described earlier, plants produce complex specialized metabolites from a relatively smaller set of central metabolic building blocks. Two types of plant defense molecules derived from secondary metabolites include phytoalexins and phytoanticipins. Phytoalexins are compounds that are produced by the plant host as a direct response to pathogen perception, whereas phytoanticipins are produced in advance of an attack and are only converted to their toxic forms post pathogen perception [102,156]. Camalexin and indole glucosinolates are two examples of these defense compound classes, respectively, that are both biosynthesized from tryptophan [100,101]. In Arabidopsis, these molecules act synergistically to provide defense against the oomycete pathogen *Phytophthora brassicae* [100]. In a metabolomics study examining necrotrophic fungal pathogens, Buxdorf et al (2013) showed that the Brassicaceae-specific fungus *Alternaria brassicicola* is more tolerant to certain glucosinolate hydrolysis products made by *Arabidopsis thaliana* than the multi-host pathogen *Botrytis cinerea* [101]. Another metabolomics-based study confirmed that canola, also a Brassica species, utilizes glucosinolates within root tissue in response to the biotrophic fungal pathogen *Plasmodiophora brassicae* that causes clubroot [102]. In addition to detecting known defense compounds, this metabolomics approach also revealed anti-fungal metabolites such as 4-methoxycyclobrassinin and dehydrocyclobrassinin that had not been previously identified in plants and could be classified as new phytoalexins [102]. 

#### 4.2.2. Volatile Organic Compounds (VOCs) 

VOCs present an interesting facet of plant defense molecules, and the very attribute of being volatile makes them perfect candidates for metabolomics analysis. As vaporous substances, VOCs are not restricted to the finite space of the pathogen invasion or colonization site(s) as is the case for some other defense compounds. Rather, these molecules are released into the surrounding environment for local as well as long-distance effects. In a recent study, plants emitting volatiles had a beneficial effect on neighboring plants subject to herbivory. Maize plants exposed to molasses grass (*Melinis minutiflora*) for a period of three weeks showed decreased egg deposition by stemborers (*Chilo partellus*) compared to maize kept in isolation [103]. Interestingly, this research group found that VOCs from the molasses grass-exposed plants contained elevated levels of compounds such as (R)-linalool, a molecule known to influence insect behavior [103,104]. In another study on switchgrass, feeding by fall armyworms (*Spodoptera frugiperda*) induced significant production of monoterpenes and sesquiterpenes—at 17% and 26% of the total VOC composition, respectively—which are compounds known to have defense activity against herbivory [157,158]. While volatile release is largely stimulated by and protects the plant from herbivory, there is mounting evidence that volatiles also serve to attract beneficial microbes [159] as well as potentially defend against bacterial pathogens by regulating stomatal aperture [105].

## 5. Case Studies

In this section, three diseases will exemplify how metabolomics elucidated central aspects of plant–pathogen interactions.

### 5.1. Soybean Cyst Nematode

Diseases caused by soybean cyst nematodes are estimated to cause an 11% annual loss of soybean yield in the United States [16]. The damage by soybean cyst nematode—*Heterodera schachtii*, which is present around the world in most producing regions [160]—led to a deficit of over 136 million bushels in 2013 [16], and represents over one billion US dollar losses each year [161,162]. The use of resistant cultivars as the main management practice is challenged by the ability of the nematodes to rapidly adaptation to overcome it [163]. The mechanisms involved in surpassing cultivar resistance are not yet fully understood. Nematodes have a biotrophic lifestyle. Juvenile staged worms infect the host roots using their stylet to mechanically penetrate tissue. Also, they secrete cell-wall degrading enzymes that do not kill the host cells [164]. Before becoming sedentary, *H. schachtii* chooses a feeding site (only one cell) which will eventually be reprogramed by complex cell signaling and be converted into a syncytium—a group of hundreds of root cells that, facilitated by cell wall degradation, are fused into one feeding structure for the parasite [165]. Host manipulation is believed to be induced by effector proteins [166], but those molecules are yet to be identified and characterized. Soybean, on the other hand, responds to nematode infection by generating reactive oxygen species and activating hormone signaling pathways to initiate defense [167,168]. For instance, in Arabidopsis, *H. schachtii* infection induces the accumulation of jasmonic acid and the decrease of abscisic acid, as well as changing the regulation of genes involved in these pathways [122]. However, auxin and ethylene have been reported to act as attractive signaling molecules for plant parasitic nematodes, directing them towards their host roots [167,169,170]. Also, *H. schachtii* produces spermine in order to interrupt reactive oxygen species formation and keep the syncytia from getting damaged [165]. Not all is bad news for soybean, since growth-promoting rhizobacteria appear to support host resistance during cyst nematode infection, as shown by Kang et al. (2018) [171]. This study integrated transcriptomics and metabolomics data to reveal that *Bacillus* sp. induce production of the phenolic compound 4-vinylphenol, which is directly related to lignin production and cell wall enhancement against pathogen penetration. Consistently, there was an up regulation of genes associated with the phenylpropanoid pathway, which is the biochemical pathway of most phenolic compounds [171]. 

### 5.2. Rice Blast Disease

Rice blast caused by *Magnaporthe oryzae*, which is the most economically serious disease in rice, is responsible for annual losses of more than 30% [172]. This fungal pathogen has a hemibiotrophic life style and infects its host at any developmental stage, causing damage to multiple tissues: Leaf, stem, node, or panicle [173]. In order to penetrate rice cells, it develops an appresorium which will lead the fungus into intracellular colonization [174]. Metabolomic analysis by GC-MS and LC-MS revealed that the fungus produces sphingolipids that are required for appresorium functionality [175]. Additionally, cell infection is thought to be tissue-specific, as the fungus possesses the genetic arsenal for specialized penetration [176]. For feeding on host cells, *M. oryzae* develops specially designed haustoria, constituting the biotrophic phase of the fungus [176]. During a compatible interaction, hyphal multiplication promotes water-soaked lesions with darker borders that are characteristic of the necrotrophic phase of the disease. To counter-attack, resistant rice genotypes produce serotonin in response to *M. oryzae* infection, leading to hypersensitive response and cell death [177] in an attempt to reduce colonization during the early biotrophic phase. Actually, rice has a complex pattern-triggered immunity response against rice blast, as pathogen recognition triggers MAPK cascades, many antimicrobial compounds are produced, hormonal signaling is stimulated, and callose deposition is induced to fortify the host cell wall [178]. A microarray study validated part of this interaction, revealing upregulation of genes related to signaling, such as MAPK and regulators associated with rice blast resistance [179]. Also, ethylene production and signaling were identified as key factors in the plant response against rice blast [93,94]. Indeed, rice lines defective in ethylene were not able to synthesize phytoalexins, important secondary metabolites related to plant defense against pathogen infection [93]. Furthermore, another study showed that not only ethylene, but other hormones like methyl jasmonate and salicylic acid were involved in the upregulation of a disease-related gene (OsERF83), which was greatly induced upon *M. oryzae* inoculation [95].

### 5.3. Bacterial Wilt in Solanaceae

*Ralstonia solanacearum* is a necrotrophic, Gram-negative, soilborne bacterium. It invades a broad host range by colonizing their xylem vessels, causing wilt and plant death. The production of extracellular polysaccharides is a prime virulence factor [92]; its accumulation inside the host xylem obstructs the vasculature and causes for wilting. GC/MS and LC/MS revealed that putrescine is produced during xylem vessel blockage in tomato plants [43]. This metabolite accelerates the disease symptoms, as it increases bacterial titers. Therefore, it is believed that *R. solanacearum* produces it, as it possesses the genetic arsenal to do so [180]. To counterattack, tomato plants produce quinic acids and flavonoids from the phenylpropanoid pathway—known for their antimicrobial activities [31,96,97,181]—and hexoses (which can act as osmotic regulators) when infected with the bacterium, as found using an untargeted metabolomics approach [98]. Hydroxycinnamic acid esters of quinic acid were identified as biomarkers of the disease, based on four cultivars that responded differently to the disease [98]. Moreover, proteomics and transcriptomics revealed that sucrose metabolism pathways were activated as an early response to *R. solanacearum* inoculation [99]. Interestingly, feruloyl-serotonin was also correlated to *Ralstonia* infection [98]. The production of this compound has been systemically associated with plant defense [182,183], even though the exact mechanisms involved are yet to be elucidated [184]. Further, spermidine synthase upregulation was correlated with higher resistance against bacterial wilt in eggplants [185]. More generally, polyamines, such as spermidine, enhance plant resistance against stress, both abiotic and biotic [186,187,188]. Because of the broad host range covered by this pathogen, biochemical markers discovered by metabolomics will continue to support the discovery of sources of resistance.

## 6. Challenges and Perspectives

One of the main challenges in host-microbe interaction studies is to discriminate between plant and pathogen metabolites. Labelling could overcome this issue, as shown by Pang et al. 2018 in Arabidopsis stomata cells inoculated with *Pseudomonas syringae* [189]. Bacterial metabolites were labelled with heavy isotopes so then they could be differentiated from the plant ones, using a targeted approach for metabolite detection. This technique enabled identification of bacterial amino and organic acids that otherwise could have been mistaken as plant products. Consequently, it was possible to demonstrate that *P. syringae* reprograms host primary metabolism and cell signaling to modulate stomatal movement [189]. This was not the first attempt to separate metabolites in this pathosystem. In 2010 Allwood et al. applied Fourier transform infrared spectroscopy to pursue differentiating Arabidopsis and *P. syringae* metabolic fingerprints [190]. This approach, which resulted in a separate analysis of plant and pathogen metabolite profiles, did not actually distinguish each compound—it detected level differences instead. Another difficulty of metabolomics—and more particularly so for plant pathology studies—is the identification of unknown compounds. There are no specific metabolite databases for plants or plant pathogens, such as those used for human [191] and yeast [192] studies. The complexity of the interaction between plant hosts and their pathogens, the intricacy of secondary metabolism—a major player in plant defense and microbial infection—certainly do not make the task of annotating metabolites any easier. Hence, identifying specialized metabolites usually involves time-consuming metabolite isolation, purification and NMR analysis or prediction-based synthesis and confirmation through matching mass transitions in LC/MS-MS. For that reason, many research institutions possess their own homemade libraries, containing metabolites often very specific to their research interests and are not always publicly available [193]. Sharing that knowledge between groups and making available the spectral information on secondary metabolites could be the most efficient way of constructing a solid and reliable plant–pathogen metabolomic database. The use of metabolomics to detect and identify pathogenic compounds synthesized during infection/colonization has increased recently, which will provide more information on the microbial side of the relationship between pathogens and their hosts. 

**Figure 4:**
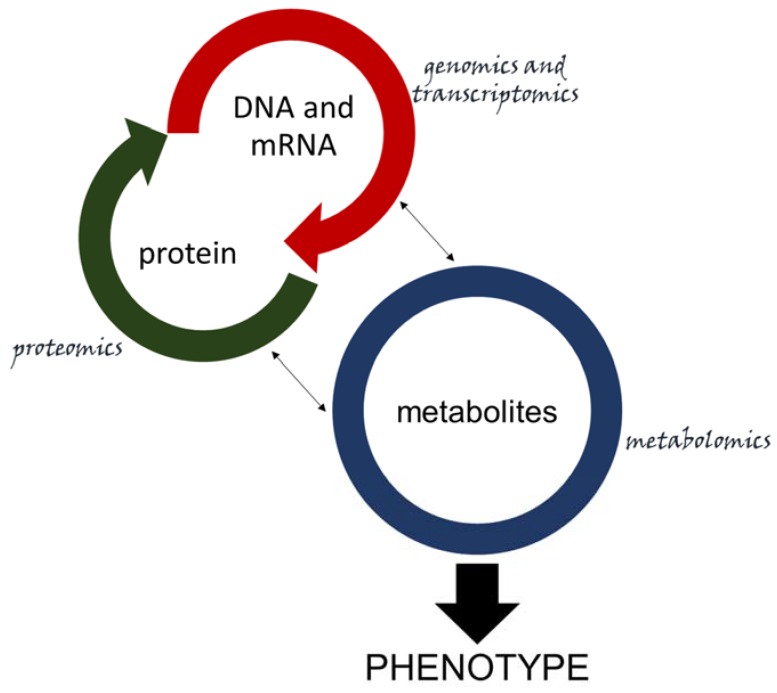
OMICs interaction. DNA and mRNA (red semi-circle) have a direct relationship with proteins (green semi-circle) and can be analyzed by genomics, transcriptomics and proteomics. Though metabolites (blue circle—which are analyzed by metabolomics) do not have a directly obvious relationship with specific genes and proteins, they are related in important ways that ultimately underlie phenotypes. The smaller arrows symbolize that indirect relationship, while the whole system is connected to phenotypes through the large arrow.

As seen extensively in this review, integration between proteomics, transcriptomics, and metabolomics is key for a deep understanding of the relationship between host and pathogen. However, integrating all the datasets from the omics is not an easy task. Linking phenotype to genotype is not as straightforward as correlating genomics data to proteomics, since there is no direct link between a gene or a set of genes and a specific metabolite [194], as it is with DNA and mRNA (hence, protein). Nonetheless, metabolites represent the final product and drive the phenotype related to the expression of many genes [64] (Figure 4). Some platforms intend to integrate omics data, but they are far from user-friendly [195], as most require advanced bioinformatics skills. Still, the software MetaboAnalyst has an appealing interface and can be used online or with R [196], and is starting to integrate some omics data with its network and pathway analysis platforms [197]. Progress in multi-omics data integration is needed to facilitate plant–microbe interaction studies. Incorporating genomic and proteomic data (which are already available in many plant pathogenic systems) to a most recently resolved metabolomic approach will require collaboration, allocation of expertise and information. This will empower researchers to build databases and platforms that will allow the combination of underlying information for a deeper understanding of host–pathogen interactions, and for developing novel approaches to improve plant resistance against biotic stress.

## Figures and Tables

**Table 1:** Summary of metabolites cited in this study involved in plant–pathogen interactions.

Role	Molecule	Function	Class	Produced by	Citation
Attack	coronatine	effector	polyketide	*Pseudomonas syringae*	[74,75]
	phenylacetic acid	toxin	organic acid	*Rhizoctonia solani*	[89]
	spermine	reactive oxygen species (ROS) interruption	polyamine	*Heterodera schachtii*	[90]
	sphingolipids	maintain appresorium functionality	lipid	*Magnaporthe oryzae*	[91]
	extracellular polysaccharides	virulence factor	polysaccharide	*Ralstonia solanacearum*	[92]
	putrescine	virulence factor	polyamine		[43]
	toxA	toxin	protein	*Pyreniphora tritici-repentis*	[38]
Defense	ethylene	cell signaling against rice blast disease	hormone	rice	[93,94]
	methyl jasmonate				[95]
	salicylic acid				
	quinic acid	defense against bacterial wilt	phenolic compound	tomato	[96,97,98,99]
	eriodictyol, kaempferol		flavonoids		
	hexoses		sugar		
	feruloyl-serotonin		hydroxyindoles		
	sarcotoxin	defense against canker	antimicrobial	transgenic citrus	[51]
	camalexin	defense against *Phytophthora brassicae*	phytoalexins/phytoantecipin	Arabidopsis	[100]
	indole glucosinolates				
		defense against *Alternaria brassicola*			[101]
		defense against *Plasmodiophora brassicae*		canola	[102]
	4-methoxyxyclobrassinin				
	dehydrocyclobrassinin				
	R-linalool	defense against insects	volatile	maize	[103,104]
	(Z)-3-hexenyl propionate	defense against *Pseudomonas syringae*	volatile	tomato	[105]
	(Z)-3-hexenyl butyrate				
